# Childhood maltreatment and major depressive disorder in well-being: a network analysis of a longitudinal community-based cohort

**DOI:** 10.1017/S0033291723000673

**Published:** 2023-11

**Authors:** Yingying Su, Muzi Li, Carl D'Arcy, Jean Caron, Xiangfei Meng

**Affiliations:** 1 Department of Psychiatry, Faculty of Medicine and Health Sciences, McGill University, Montreal, QC, Canada; 2 Douglas Research Centre, Montreal, QC, Canada; 3 School of Public Health, University of Saskatchewan, Saskatoon, SK, Canada; 4 Department of Psychiatry, College of Medicine, University of Saskatchewan, Saskatoon, SK, Canada

**Keywords:** Childhood maltreatment, Life satisfaction, Major depressive disorder, Network analysis, Psychological well-being, Quality of life

## Abstract

**Background:**

Little has been done to comprehensively study the relationships between multiple well-being constructs at a time. Even less is known about whether child maltreatment and major depressive disorder (MDD) impact different well-being constructs. This study aims to examine whether maltreated or depressed individuals have specific impacts on well-being structures.

**Methods:**

Data analyzed were from the Montreal South-West Longitudinal Catchment Area Study (*N* = 1380). The potential confounding of age and sex was controlled by propensity score matching. We used network analysis to assess the impact of maltreatment and MDD on well-being. The centrality of nodes was estimated with the ‘strength’ index and a case-dropping bootstrap procedure was used to test network stability. Differences in the structure and connectivity of networks between different studied groups were also examined.

**Results:**

Autonomy and daily life and social relations were the most central nodes for the MDD and maltreated groups [MDD group: *strength coefficient* (*SC*)*_autonomy_* = 1.50; *SC_daily life and social relations_* = 1.34; maltreated group: *SC_autonomy_* = 1.69; *SC_daily life and social relations_* = 1.55]. Both maltreatment and MDD groups had statistical differences in terms of the global strength of interconnectivity in their networks. Network invariance differed between with and without MDD groups indicating different structures of their networks. The non-maltreatment and MDD group had the highest level of overall connectivity.

**Conclusions:**

We discovered distinct connectivity patterns of well-being outcomes in maltreatment and MDD groups. The identified core constructs could serve as potential targets to maximize the effectiveness of clinical management of MDD and also advance prevention to minimize the sequelae of maltreatment.

## Introduction

Well-being includes hedonic (Kahneman, Diener, & Schwarz, 1999) and eudemonic well-being (Ryff & Singer, 2008). Hedonic well-being refers to pleasurable experiences and minimizing suffering through the pursuit of goals or valued outcomes to achieve a higher level of well-being (Vanhoutte, 2014). Whereas eudemonic well-being relates to living well and fully, and the realization of human potential which focuses on meaning, self-realization, and flourishing in life (Keyes, 2002; Ryan, Huta, & Deci, 2008). Hedonic well-being can be seen as subjective well-being consisting of life satisfaction (LS) and positive emotions (Diener, 2009). LS is an overall appraisal of one's life as a whole (Diener, 1984). Eudemonic well-being focuses on self-acceptance, positive relationships with others, personal growth, purpose in life, environmental mastery, and autonomy. Ryff developed a concept of psychological well-being (PWB) to assess eudemonic well-being (Ryff & Keyes, 1995). Likewise, quality of life (QoL) is an important aspect of eudemonic well-being and has been developed as a targeted measure of health research (Hyde, Wiggins, Higgs, & Blane, 2003). QoL is defined as individuals' perception of their position in life in the context of the culture and value systems in which they live and in relation to their goals, expectations, standards, and concerns (Whoqol Group, 1998). While QoL, PWB, and LS are subdomains of well-being (Anand & Arora, 2009), each of them has distinct elements and reflects distinguishing features (Clare et al., 2014). A holistic evaluation including measures for both hedonic and eudemonic aspects is essential for a comprehensive understanding of the concept of well-being.

The literature has cumulated ample evidence on the negative consequences of childhood maltreatment (CM) and major depressive disorder (MDD) on subsequent well-being (Edwards, Holden, Felitti, & Anda, 2003; Engel, Chen, Richardson, & Mihalopoulos, 2018; Su, D'Arcy, & Meng, 2020). Individuals suffering from MDD reported substantially lower PWB, poorer QoL, and decreased LS than their counterparts (Koivumaa-Honkanen, Kaprio, Honkanen, Viinamäki, & Koskenvuo, 2004; Nierenberg et al., 2010). Edmondson and MacLeod (2015) found that depressed individuals were more likely to have a diminished resource for a wide range of aspects of well-being, particularly positive relationships with others. However, the correlations were not as strong as expected and MDD disproportionally affected different dimensions of well-being (Ruini et al., 2003). CM is one of the most important risk factors for subsequent MDD (Li, D'Arcy, & Meng, 2016). Studies have shown that CM is associated with remarkable functional and structural brain changes observed later in adulthood among those without psychopathology (Dannlowski et al., 2012). Like MDD, CM also predicts poor well-being and impairment in later life (Cohen, Brown, & Smailes, 2001). Fergusson, McLeod, and Horwood (2013) discovered a significant association between CM and LS in a 30-year New Zealand birth cohort study. Other studies have shown that adult survivors of CM reported significant and sustained impairments in QoL (Weber, Jud, & Landolt, 2016) and poorer PWB (Arslan, 2021; Kong, 2018). It is possible that CM could affect one's well-being without having psychopathology, and CM and MDD may have different impacts on well-being. However, fewer studies have been conducted to comprehensively investigate multiple dimensions of well-being among individuals exposed to CM or living with MDD. Even less is known about those exposed to CM and had MDD, it is crucial to assess the combined effect of CM and MDD on their well-being.

Given CM and MDD negatively influence well-being, it is of clinical importance and public health priority to specify the impact of CM and MDD on well-being. A closer look at which dimensions of well-being are affected by CM and/or MDD would shed constructive light on selective and targeted interventions aimed at well-being improvements (Diener, 2000). More importantly, a systematic examination of complex relationships among diverse measures of well-being provides unique opportunities to comprehensively explore commonalities and divergences of hedonic and eudemonic well-being among diversified populations.

Most previous studies targeted on a specific well-being measure, rather than working with multiple well-being measures at a time (Afifi et al., 2007; Corso, Edwards, Fang, & Mercy, 2008; Greger, Myhre, Lydersen, & Jozefiak, 2016). The conventional analytical approaches cannot assess the relationships between multiple well-being measures in a single analysis. For example, factor analysis is frequently used to assess the latent dimensions of a set of variables. It cannot provide the information on the relationships between these dimensions (Van de Weijer, Landvreugd, Pelt, & Bartels, 2021). Borsboom proposed a network theory to estimate the jointed effect of multiple factors in mental health and suggested a network analysis to analyze the relationships between these factors at one time in a single analysis (Borsboom & Cramer, 2013). Because experiences of CM and MDD are likely to affect multiple well-being domains, network analysis could be adopted to simultaneously investigate complex relationships between PWB, QoL, and LS across different populations. The network analysis could test interactions between well-being measures and show complex patterns of their relationships. This network approach could also identify components that are central to the network indicating the most connected component affecting the rest of components in the network (Epskamp, Kruis, & Marsman, 2017). Furthermore, bridge nodes and critical edges implicate the strongest links in the network (Jones, Ma, & McNally, 2021). These are important elements in the network models and have clinical implications in identifying effective interventions for maximum payoff.

It is not uncommon to apply the network approach to mental disorders. For instance, studies have applied the network approach to illustrate associations between CM and psychopathology (Monteleone et al., 2019; Rodgers et al., 2019). However, these findings are mainly focused on the distinct psychopathology profiles between those with and without CM exposures. Well-being is an important component of effective clinical management and prevention for individuals suffering from psychopathology. Improving well-being has significant clinical and practical values for those with exposure to CM or MDD.

To fill the important knowledge gap in well-being, the present study aimed to examine whether individuals exposed to CM or suffering from MDD had unique and diversified structures of well-being by systematically analyzing a longitudinal community-based population cohort. By assessing relationships of multiple well-being measures, we can contrast how different well-being measures interconnected and identify the key constructs in the network of well-being. Differences in their networks of well-being between CM and non-CM groups as well as depressed and non-depressed groups would be also tested to examine if CM and MDD had differential impacts on these networks. We hypothesized that those with CM even in the absence of MDD and/or MDD not only reported poorer well-being but also had differential structures of well-being networks.

## Method

### Study cohort

Data analyzed were from the Zone d'Épidémiologie Psychiatrique du Sud-Ouest de Montréal (ZEPSOM), which is a longitudinal community-based cohort study of a representative sample of five neighborhoods in the South-West sector of Montreal, Canada. ZEPSOM cohort is a random sample of area residents aged 15–65 years old, which was followed at 2-year intervals starting from the baseline year of 2007 (Caron et al., 2012). The present study sample (*N* = 1380) is restricted to study respondents who completed their interviews at wave 4 and wave 5 of the cohort and had information on CM, MDD, and well-being outcomes. Online Supplementary Fig. S1 presents a summary of the sample size across each data collection. Details on survey methods have been described previously (Caron et al., 2012).

### Measures

#### Childhood maltreatment (CM)

CM was assessed using the Childhood Trauma Questionnaire (CTQ) (Bernstein & Fink, 1998). CTQ is a 28-item self-reported questionnaire that assesses different types of CM, specifically emotional abuse, physical abuse, sexual abuse, emotional neglect, and physical neglect. Responses to each item were rated on a five-point Likert scale, ranging from 0 (never true) to 5 (always true). The cutoff points for each category used in the present study were based on Bernstein, Fink, Handelsman, and Foote (1998).

#### Major depressive disorder (MDD)

MDD was assessed by the WHO's World Mental Health (WMH) 2000 project Composite International Diagnostic Inventory (CIDI) (Kishore, Kapoor, & Reddaiah, 1999). It is a fully structured diagnostic interview assessing MDD using the definitions and criteria of the Diagnostic and Statistical Manual of Mental Disorders, 4th edition (DSM-IV) and the International Statistical Classification of Diseases and Related Health Problems, 10th revision (ICD-10) (American Psychiatric Association, 2000; Zivetz, 1992).

#### Well-being outcomes

*Psychological well-being* (Ryff & Keyes, 1995) was assessed using the subscale of Mental Health Continuum-Short Form (MHC-SF) (Keyes, 2005) and was validated in Canada by Doré, O'Loughlin, Sabiston, and Fournier (2017). It measures several dimensions: *autonomy*, *environmental mastery*, *personal growth*, *positive relations with others*, *purpose in life*, and *self-acceptance*. Response categories were coded as ‘never’ (0), ‘once or twice’ (1), ‘about once a week’ (2), ‘about two or three times a week’ (3), ‘almost every day’ (4), and ‘every day’ (5). Its Cronbach's *α* coefficient was 0.85.

*Quality of life* was assessed with the Satisfaction with Life Domains Scale (SLDS), which is a brief self-reported measure of QoL that was developed by Andrews and Withey in (1976) and was adapted by Baker and Intagliata (1982) for psychiatric patients. It was validated in Canada by Caron, Mercier, and Tempier (1997) on several populations. It had 20 items, measured with a seven-point Likert scale, and grouped into five subscales: *daily life and social relations*, *living environment*, *autonomy*, *relationships*, and *leisure activities*. The internal consistency was excellent, the Cronbach's *α* is 0.92 for the whole scale and the range of Cronbach's *α*s varies from 0.72 to 0.84.

*Life satisfaction* was measured by the subscale of Personal Well-being Index of the Australian Unity Well-being Index which is based on the average levels of satisfaction with various aspects of personal life (Cummins, Eckersley, Pallant, Van Vugt, & Misajon, 2003). The scale has items that address satisfaction with health, living standards, what one has achieved in life, security, the groups of people one is part of, security about the future and relations with others, and spirituality or religion. Respondents were asked to rate their satisfaction on a scale of 0 (extremely dissatisfied) to 10 (extremely satisfied). Its Cronbach's *α* value in this study was 0.85.

### Statistical analysis

To minimize the potential confounding of sex and age in the relationships between CM and well-being outcomes, propensity score matching (PSM) combined with the inverse probability-weighted regression-adjustment (IPWRA) analyses were firstly conducted. IPWRA was chosen to emulate the covariate balance typically achieved by a randomized study where differences are due to chance (Austin, 2011). The average treatment effect on the treated (ATET) was used in the present study to quantify the effects of CM and MDD on three outcomes: PWB, QoL, and LS.

The network analysis was then conducted to model the well-being structures. The R (v.4.1.1) *qgraph* (Epskamp, Cramer, Waldorp, Schmittmann, & Borsboom, 2012) and *bootnet* packages (Epskamp, Borsboom, & Fried, 2018) were used. Gaussian Graphical Models (GGMs) were then used to visualize the network structures, in which edge weights represented partial correlation coefficients between nodes (Costantini et al., 2015). The graphical LASSO (Least Absolute Shrinkage and Selection Operator) was applied to regularize the magnitude of each regression weight and set small coefficients to zero (Friedman, Hastie, & Tibshirani, 2008). The degree of shrinkage and selection operator were determined by the extended Bayesian Information Criterion (EBIC) (Epskamp et al., 2018). The networks were visualized using the Fruchterman–Reingold algorithm, which placed nodes with stronger and more frequent associations with one another together (Epskamp et al., 2018). Even though three measures of node centrality – strength, closeness, and betweenness are often used to explore the relative importance of each item in the network analysis, only strength was used to interpret the findings of the present study as researchers have noted that neither betweenness nor closeness is a reliable measure of network structure (Bringmann et al., 2019). Strength is an overall connection of a node with other nodes in the network and is the most straightforward and frequently used centrality index (Hevey, 2018). Furthermore, the stability of strength was assessed by the correlation stability (CS) coefficient which was computed by case-dropping bootstrapping with 1000 samples (Costenbader & Valente, 2003). It calculated the maximum proportion of cases that can be dropped while maintaining a correlation of above 0.7 between the centrality indices of the original dataset and subsets with a 95% probability.

Finally, we used the network comparison test (NCT) to identify differences in the overall structures of networks and to compare the cumulative strength of the connections (edges) within the networks among different study groups using the R package *NetworkComparisonTest* (Van Borkulo et al., 2022). The NCT is a permutation-based hypothesis test that analyzes differences in the global strength and structure between two networks *via* repeated samples of bootstrapped individuals (Van Borkulo et al., 2015). Comparisons of edge strength were done by testing the difference in the strength of a specific edge with corrections using Holm–Bonferroni methods for multiple comparisons.

## Results

### Characteristics of the study cohort

The study cohort included a total of 1380 participants aged between 17 and 79 years with a mean age of 50.6 years (s.d. 13.9). Among all the studied participants, 61.6% (*N* = 850) of the participants were females and 38.4% (*N* = 530) were males. Less than half of the sample was single (*N* = 595, 43.1%) and almost thirds (*N* = 520, 37.7%) were married or in common-law relationships, and the rest (*N* = 265, 19.2%) were divorced or separated or widowed. Most participants (*N* = 1256, 91.0%) had completed post-high school graduation, with only 5.4% (*N* = 50) of participants completing high school and 3.6% (*N* = 74) having less than a high school education. Mean, standard deviation, skewness, kurtosis, and frequency of the studied outcomes are reported in online Supplementary Table S1.

### The impact of CM and MDD on well-being

A total of 1352 participants were matched based on their CM by the PSM analysis using nearest-neighbor matching methods. The balanced propensity was found satisfactory. Online Supplementary Table S2 presents the average effects of CM on three studied outcomes. Compared to those without exposure to CM, those exposed to CM were 1.71 (95% CI −2.36 to −1.06) points lower in PWB, 7.10 (95% CI −8.87 to −5.33) points lower in QoL, and 2.35 (95% CI −3.01 to −1.69) points lower in LS after taking sex and age into account. In addition, we also examined the influence of specific CM types on three outcomes (Table 1). Consistently, emotional neglect and emotional abuse had stronger impacts on all three outcomes. Specifically, emotional neglect and abuse were associated with lower PWB (ATET_neglect_ = −2.49, 95% CI −3.13 to −1.84; ATET_abuse_ = −2.04, 95% CI −2.76 to −1.32), QoL (ATET_neglect_ = −9.17, 95% CI −10.90 to −7.45; ATET_abuse_ = −8.72, 95% CI −10.65 to −6.79), and LS (ATET_neglect_ = −2.88, 95% CI −3.53 to −2.23; ATET_abuse_ = −2.61, 95% CI −3.32 to −1.87). We also matched participants based on their MDD status, a total of 1365 participants were matched. The balanced propensity for MD was also found to be satisfactory. Likewise, those with MDD reported scores 2.87 (95% CI −3.72 to −2.02), 8.75 (95% CI −11.10 to −6.39), and 3.81 (95% CI −4.65 to −2.97) points lower on the PWB, QoL, and LS measures, respectively.

**Table 1.** Impacts of subtypes of childhood maltreatment on well-being

Well-being	Maltreatment subtypes	ATET	Robust s.e.	*p* value	95% CI
Psychological wellbeing	Emotional abuse	−2.04	0.37	<0.001	−2.76 to −1.32
	Physical abuse	−1.04	0.41	0.011	−1.84 to −0.24
	Sexual abuse	−1.28	0.34	<0.001	−1.95 to −0.61
	Emotional neglect	−2.49	0.33	<0.001	−3.13 to −1.84
	Physical neglect	−1.34	0.40	0.001	−2.13 to −0.56
Quality of life	Emotional abuse	−8.72	0.98	<0.001	−10.65 to −6.79
	Physical abuse	−6.41	1.12	<0.001	−8.61 to −4.21
	Sexual abuse	−5.86	0.96	<0.001	−7.73 to −3.98
	Emotional neglect	−9.17	0.88	<0.001	−10.90 to −7.45
	Physical neglect	−7.43	1.09	<0.001	−9.58 to −5.28
Life satisfaction	Emotional abuse	−2.61	0.38	<0.001	−3.32 to −1.87
	Physical abuse	−1.83	0.42	<0.001	−2.67 to −1.00
	Sexual abuse	−1.82	0.36	<0.001	−2.53 to −1.10
	Emotional neglect	−2.88	0.33	<0.001	−3.53 to −2.23
	Physical neglect	−2.38	0.42	<0.001	−3.20 to −1.56

Note: ATET, average treatment effect on the treated; s.e., standard error; CI, confidence interval; sex and age were adjusted in the model.

### The network structures of well-being among CM and non-CM groups

**Fig. 1.**
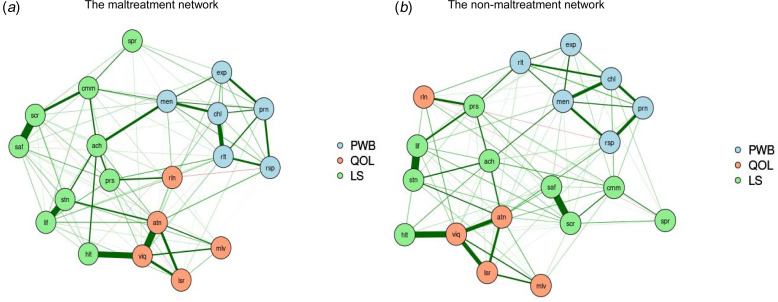
The network structures of well-being among individuals with and without exposure to childhood maltreatment. *Notes*: PWB, psychological well-being; QOL, quality of life; LS, life satisfaction; prn, liked personality; rsp, responsibilities management; rlt, warm and trusting relationships; chl, challenge for becoming better; exp, express opinions; men, life meaning; mlv, housing-neighborhood; viq, daily life and social relations; rln, personal relationships; atn, autonomy; lsr, spare time activities; lif, satisfaction with life; stn, satisfaction with living standard; hlt, satisfaction with health; ach, satisfaction with achievement; prs, satisfaction with personal relationship; saf, satisfaction with safety; cmm, satisfaction with community; scr, satisfaction with security; spr, satisfaction with spirituality.

Figure 1 presents the estimated networks of the studied outcomes for CM and non-CM groups. The network of the CM group consisted of 216 non-zero edges out of 400 edges with 54.0% density, whereas the network of the non-CM group consisted of 246 non-zero edges out of 400 edges with 61.5% density. In the CM network, the nodes with the highest strength centrality were *autonomy*, *daily life and* so*cial relations*, and *warm and trusting relationships*. Whereas *warm and trusting relationships*, *daily life and social relations*, and *satisfaction with security* were the nodes with the highest strength centrality for the non-CM network (online Supplementary Fig. S2). Stability analyses of CM and non-CM networks observed sufficient CS coefficient for strength (CS_CM_ = 0.75; CS_NOCM_ = 0.60), indicating that the node strength in these two networks was accurately estimated.

### Comparison between CM and non-CM groups networks structure

The NCT identified significant differences in global strength (*S* = 1.00, *p* = 0.02) between the two networks but no difference in network invariance (*M* = 0.18, *p* = 0.48) for CM and non-CM groups. Even though the structure of these two networks was not statistically different, the strength of the connections within the network was greater for non-maltreated individuals (global strength = 10.66) than for maltreated individuals (global strength = 9.66). When each specific subtype of CM was examined, no significant differences in network strength and structure were observed in comparison with non-maltreated individuals (online Supplementary Table S3 and Fig. S3).

### The network structure of well-being for MDD and non-MDD groups

**Fig 2.**
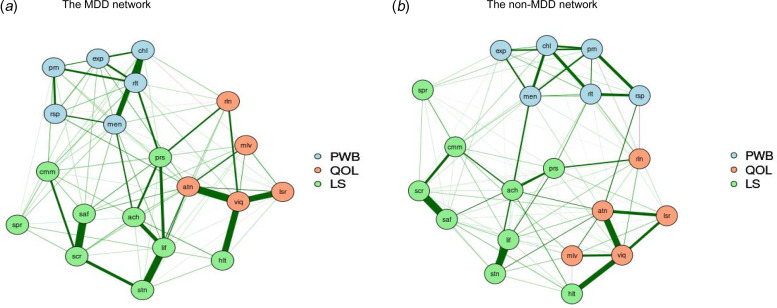
The network structures of well-being among individuals with and without MDD. *Notes*: MDD, major depressive disorder; PWB, psychological wellbeing; QOL, quality of life; LS, life satisfaction; prn, liked personality; rsp, responsibilities management; rlt, warm and trusting relationships; chl, challenge for becoming better; exp, express opinions; men, life meaning; mlv, housing-neighborhood; viq, daily life and social relations; rln, personal relationships; atn, autonomy; lsr, spare time activities; lif, satisfaction with life; stn, satisfaction with living standard; hlt, satisfaction with health; ach, satisfaction with achievement; prs, satisfaction with personal relationship; saf, satisfaction with safety; cmm, satisfaction with community; scr, satisfaction with security; spr, satisfaction with spirituality.

Figure 2 presents the estimated networks for MDD and non-MDD groups. The network of the depressed group consisted of 190 non-zero edges out of 400 edges with 47.5% density, whereas the network of non-depressed group consisted of 256 non-zero edges (out of 400 edges) with 64.0% density. The nodes with the highest strength centrality were slightly different between the depressed and the non-depressed groups. In the network of depressed individuals, these nodes were *autonomy*, *daily life and social relations*, and *satisfaction with safety*, whereas *daily life and social relations*, *warm and trusting relationships*, and *satisfaction with life* were the nodes with the highest strength centrality in the non-depressed network (online Supplementary Fig. S4). The stability of the centrality indices was sufficient, as the CS coefficients of the node strength centrality were 0.67 and 0.75 for the depressed and the non-depressed groups.

### Comparison between MDD and non-MDD groups networks structure

The NCT test showed significant differences in the global strength between MDD and non-MDD networks (*S* = 1.23, *p* = 0.02), indicating that the strength of the connections within the network was greater for non-depressed individuals (global strength = 10.42) than for depressed individuals (global strength = 9.19). We also found a significant difference between depressed and non-depressed groups networks (*M* = 0.26, *p* ⩽ 0.01), which suggests that the overall structures of these two networks were different. Post hoc tests were then conducted to identify individual edges (connections) that differed between these two networks. Three edges differed significantly in the depressed and non-depressed networks. In the MDD network, there were positive connections between satisfaction with life and satisfaction with achievement, satisfaction with living standard and satisfaction with security, as well as warm and trusting relationships and satisfaction with the community (*p* ⩽ 0.05). In contrast, these edges disappeared or decreased in the non-MDD network.

### The effects of CM and MDD on well-being network

**Fig. 3.**
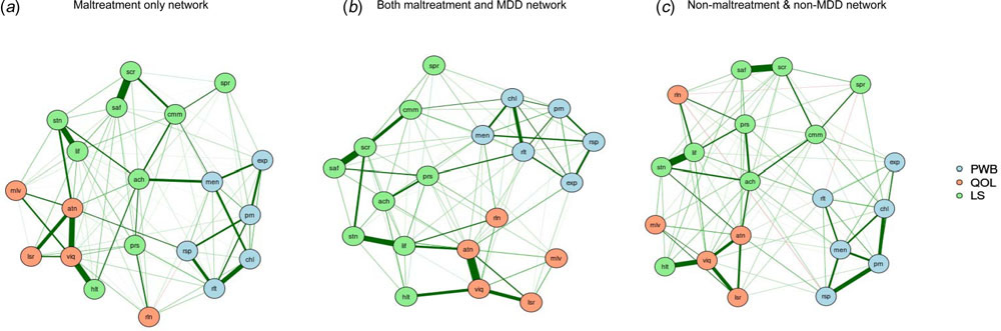
The network structures of well-being among individuals in the maltreatment only group, non-maltreatment/MDD group, and both maltreatment and MDD group *Note*s: MDD, major depressive disorder; PWB, psychological wellbeing; QOL, quality of life; LS, life satisfaction; prn, liked personality; rsp, responsibilities management; rlt, warm and trusting relationships; chl, challenge for becoming better; exp, express opinions; men, life meaning; mlv, housing-neighborhood; viq, daily life and social relations; rln, personal relationships; atn, autonomy; lsr, spare time activities; lif, satisfaction with life; stn, satisfaction with living standard; hlt, satisfaction with health; ach, satisfaction with achievement; prs, satisfaction with personal relationship; saf, satisfaction with safety; cmm, satisfaction with community; scr, satisfaction with security; spr, satisfaction with spirituality.

To further assess the independent impacts of CM and MDD on well-being outcomes, we compared the networks among individuals with CM only, with both CM and MDD, and those without either CM or MDD (Fig. 3). There were respectively 232, 202, and 176 non-zero edges for the non-CM and MDD group, the CM-only group, and with CM and MDD group. Likewise, for people with CM only, the nodes with the highest strength centrality were *daily life and social relations*, *autonomy*, and *warm and trusting relationships*. For those in the non-CM and MDD group, the strongest nodes were *warm and trusting relationship*s, *satisfaction with life*, and *satisfaction with security*. For those with CM and MDD, *autonomy*, *satisfaction with security*, and *satisfaction with life* were the strongest nodes (online Supplementary Fig. S5). Stability analyses observed sufficient CS coefficient for strength in the CM-only network (CS = 0.75), with CM and MDD network (CS = 0.75), and non-CM and MDD network (CS = 0.52), indicating the order of node strength is interpretable with care.

NCT findings replicated the findings on the independent impact of CM on well-being outcomes. There were significant differences in global strength (*S* = 0.97, *p* = 0.01) but no difference in network invariance (*M* = 0.16 *p* = 0.82) for CM only and non-CM and MDD networks. The studied outcomes were densely connected among individuals in the non-CM and MDD group (global strength = 9.80) compared to those with CM only (global strength = 8.83). Similarly, significant differences in global strength (*S* = 2.72, *p* = 0.01) but no difference in network invariance (*M* = 0.22 *p* = 0.63) were found between networks of those with both CM and MDD (global strength = 8.81), and those in the non-CM and MDD group (global strength = 11.53), suggesting that non-CM and MDD network had a greater density in the studied outcomes compared to both CM and MDD groups. However, no statistical difference in terms of network strength (*S* = 0.29, *p* = 0.59) or structure (*M* = 0.25 *p* = 0.17) was found in the CM-only and with both CM and MDD groups. The details are available in online Supplementary Table S4.

## Discussion

The present study provides one of the first pieces of evidence on the specific and unique impacts of CM and MDD on well-being outcomes in a matched community-based cohort. Our findings offer robust evidence to uncover diverse patterns of connections between multiple constructs of well-being. Consistently, we found that both CM and MDD were associated with a statistically significant decrease in well-being. The CM group and the non-CM group had statistical differences in the strengths of connections between the studied well-being constructs but not how they connected to each other. In contrast, MDD and non-MDD groups had substantial differences in terms of the structures of studied constructs, how they connected to each other, as well as the density of network connectivity. Furthermore, we found the group with both CM and MDD had a lower density among the studied constructs compared with the non-CM/MDD group and the CM-only group.

In line with the literature (Arslan, 2021; Kong, 2018), we found that CM was associated with reduced ratings of all the studied outcomes after controlling for sex and age. Compared to other subtypes of CM, emotional neglect and abuse had stronger associations with the studied outcomes. This is consistent with the findings from a systematic review and meta-analysis, which found emotional abuse and neglect approximately doubled the risk of adverse long-term health outcomes (Norman et al., 2012). Likewise, the negative impact of MDD on the studied outcomes was also observed. Neurobiological disruptions altered by CM in the early years of life and/or MDD during later life can in turn negatively affect physical, cognitive, emotional, and social functioning leading to psychological, behavioral, and even physical problems throughout the life span (Smith & Pollak, 2020). Such problems substantially influence how people perceive their health.

For the network of well-being outcomes, the most central nodes in the CM group were QoL items (autonomy and daily life and social relations) followed by PWB items (warm and trusting relationships). These items were more important compared to other nodes as they were strongly connected to others. CM intervention and prevention have pointed out the need of dealing with long-lasting negative consequences that affect the daily lives of CM victims, and QoL can be used as a critical indicator of living a good and fulfilling life (Greger et al., 2016). In line with the self-determination theory, which suggests that people are motivated to grow and change by three innate and universal psychological needs (autonomy, competence, and connection) (Ryan & Deci, 2000), we discovered that autonomy was centered in the networks among maltreated victims. We also found that positive relationships with others played an important role in the studied outcomes. This finding was also noted in Ryff and Singer's review of PWB (Ryff & Singer, 1996). These central nodes can trigger, develop, and maintain states of PWB in the whole network (McNally, 2016). Targeted interventions for enhancing well-being should explicitly focus on these indicators to improve health and well-being benefits.

Our network analysis identified the differences in network density, but not in network structure between CM and non-CM group networks. The connections between constructs in the non-CM group network were stronger than those in the CM group network. The intense density in the studied outcomes demonstrated fewer health issues and better well-being. These findings are consistent with existing studies on the negative correlations between CM and lower levels of LS, PWB, and QoL (Ustuner Top & Cam, 2021; Xiang, Yuan, & Zhao, 2021). Taken together, the present study further advances our knowledge of how CM affects overall well-being and advocates a novel perspective to deliver more targeted interventions on specific indicators of well-being among maltreated victims.

For those with MDD, QoL (autonomy and daily life and social relations) and satisfaction with safety were the most central nodes in their well-being network. Another study also found that satisfaction within specific elements of life played an important role in the well-being network (Blasco-Belled & Alsinet, 2021). Given almost all the constructs were positively interrelated, we speculate that enhancing QoL and LS would intensify the connectivity within the network and promote higher levels of other constructs of well-being. In addition, we found that the non-MDD network was more densely interconnected than the MDD network, suggesting that MDD was associated with weaker connections between constructs of well-being. This is consistent with the findings of MDD associated with lower levels of QoL, PWB, and LS (Gigantesco et al., 2019; Lantheaume, Fernandez, Lantheaume, Moták, & Conceição, 2022; Nes, Czajkowski, Roysamb, Reichborn-Kjennerud, & Tambs, 2008).

To identify the independent impact of the CM without the presence of the MDD, and whether CM and MDD had differential impacts on well-being, we tested the differences in global connectivity of nodes in the network among the CM-only group, the CM and MDD group, and the non-CM and MDD group. QoL nodes remained more influential in the networks of the CM-only group and the CM and MDD group rather than in the non-CM and MDD group. QoL was more strongly connected with other nodes and had a greater impact on other nodes in the network. We found that compared to the CM-only or the CM and MDD group, the non-CM and MDD group had a higher level of overall connectivity in their network structure. These findings highlight the predisposition of CM victims in well-being even though no MDD was diagnosed. Our findings offer psychological and emotional evidence to expand the neurobiological findings of the ‘limbic scar’ among CM victims for enduring consequences of CM exposures on their functional and structural alternations in the brain (Dannlowski et al., 2012). These networks add meaningful and novel evidence to present a holistic view of the comprehensive influences of CM on well-being (Muntean et al., 2022).

Our study has several practical and clinical implications. First, this study reiterates the close relationships between CM/MDD and well-being and discovers important constructs in well-being. Second, the core constructs of well-being should be prioritized in the interventions for people with MDD or exposures to CM. These constructs would maximize the effectiveness of the clinical management of MDD as well as minimize the negative consequences of CM. Integrative treatment programs could also take PWB, QoL, and LS as indicators of effective treatment.

The study has several limitations to be noted. First, CM was assessed by a self-reported scale, even though CTQ is a well-accepted scale to measure CM. The retrospective measure of CM might lead to potential recall bias and produce spurious results. Second, the present study focused on three studied outcomes. It is possible to have different networks of well-being when different constructs are included. Future research is warranted to replicate and extend these current findings in different combinations of well-being measures and study populations. Third, network analyses were not conducted for different subgroups of CM due to the limited sample sizes in each subgroup as the statistical power was not sufficient to estimate the stability of strength centrality. Finally, cross-sectional networks face the limitation that they may only reveal the co-occurrence of each node but not how they follow each other over time (Bos et al., 2017). Longitudinal studies with repeated measures are warranted to assess the temporal dynamics of relationships between CM/MDD and well-being.

## Conclusions

The present study triangulated the structures and dynamics of well-being in relation to CM and MDD and delineated the differences in the networks of well-being across CM and MDD groups. Multiple well-being measures were more densely connected in the non-CM and MDD group than in the CM-only group as well as the group with both CM and MDD. These findings highlight the necessity of identifying the exposures to CM as it is tied to adverse well-being even in the absence of MDD. The identified most influential nodes in each network suggest the most important interrelationships induced by CM or MDD. These core elements of well-being outcomes could be targeted to maximize the effectiveness of clinical management of MDD as well as prevention efforts to minimize the negative consequences of CM.

## Supporting information

Su et al. supplementary material Su et al. supplementary material

## Supplementary material

The supplementary material for this article can be found at https://doi.org/10.1017/S0033291723000673
