# Supplementary material for: Childhood maltreatment and major depressive disorder in well-being: a network analysis of a longitudinal community-based cohort
Source: Psychol Med. 2023 Mar 24;53(15):7180–8. doi: 10.1017/S0033291723000673 (PMC10719668; doi:10.1017/S0033291723000673)

| **Table S1**. Mean, Standard Deviation, Minimum, Maximum, Skewness, and Kurtosis of the studied well-being measures. | | | | | | |
| --- | --- | --- | --- | --- | --- | --- |
| Items | Min | Max | Mean | SD | Skewness | Kurtosis |
| Liked personality | 0 | 5 | 3.70 | 1.13 | -1.20 | 1.32 |
| Responsibilities management | 0 | 5 | 4.05 | 1.09 | -1.39 | 1.88 |
| Warm and trusting relationships | 0 | 5 | 4.01 | 1.05 | -1.42 | 2.16 |
| Challenge for becoming better | 0 | 5 | 3.56 | 1.28 | -0.99 | 0.40 |
| Express opinions | 0 | 5 | 3.95 | 1.14 | -1.33 | 1.62 |
| Life meaning | 0 | 5 | 3.85 | 1.23 | -1.36 | 1.46 |
| Neighborhood | 5 | 21 | 16.75 | 2.61 | -0.72 | 0.86 |
| Daily life & social relations | 14 | 41 | 33.76 | 4.34 | -0.69 | 0.72 |
| Personal relationships | 2 | 21 | 15.81 | 4.35 | -0.76 | -0.39 |
| Autonomy | 10 | 42 | 33.25 | 4.97 | -0.87 | 1.29 |
| Spare time activities | 2 | 14 | 10.64 | 2.14 | -0.73 | 1.03 |
| Satisfaction with life | 0 | 3 | 2.03 | 0.67 | -0.22 | -0.17 |
| Satisfaction with living standard | 0 | 3 | 1.96 | 0.73 | -0.14 | -0.64 |
| Satisfaction with health | 0 | 3 | 1.91 | 0.80 | -0.07 | -0.96 |
| Satisfaction with achievement | 0 | 3 | 1.99 | 0.72 | -0.12 | -0.70 |
| Satisfaction with personal relationship | 0 | 3 | 2.13 | 0.75 | -0.33 | -0.82 |
| Satisfaction with safety | 0 | 3 | 2.35 | 0.71 | -0.67 | -0.54 |
| Satisfaction with community | 0 | 3 | 1.86 | 0.76 | 0.05 | -0.89 |
| Satisfaction with security | 0 | 3 | 2.04 | 0.78 | -0.21 | -0.99 |
| Satisfaction with spirituality | 0 | 3 | 1.91 | 0.89 | 0.01 | -1.40 |

Note: Min: minimum; Max: maximum; SD: standard deviation.

| \| **Table S2**. The impact of CM and MDD on well-being. \| \| \| \| \| \| \| \| --- \| --- \| --- \| --- \| --- \| --- \| --- \| \| Outcomes \| CM \| \| \| \| MDD \| \| \| ATET \| Robust SE \| 95% CI \| ATET \| Robust SE \| 95% CI \| \| Psychological well-being \| -1.71 \| 0.33 \| -2.36, -1.06 \| -2.87 \| 0.43 \| -3.72, -2.02 \| \| Quality of life \| -7.10 \| 0.90 \| -8.87, -5.33 \| -8.75 \| 1.20 \| -11.10, -6.39 \| \| Life satisfaction \| -2.35 \| 0.34 \| -3.01, -1.69 \| -3.81 \| 0.43 \| -4.65, -2.97 \|   Notes: CM: childhood maltreatment; MDD: major depressive disorder; ATET: Average treatment effect on the treated; SE: Standard error; CI: Confidence interval; sex and age were adjusted in the model. | | | | | |
| --- | --- | --- | --- | --- | --- | --- | --- | --- | --- | --- | --- | --- | --- | --- | --- | --- | --- | --- | --- | --- | --- | --- | --- | --- | --- | --- | --- | --- | --- | --- | --- | --- | --- | --- | --- | --- | --- | --- | --- | --- | --- | --- | --- | --- | --- | --- |
| **Table S3**. Permutation results of network comparison test across various subtypes of CM and non-CM groups. | | | | | |
|  | EA | PA | SA | EN | PN |
| Network invariance | 0.20 | 0.20 | 0.17 | 0.19 | 0.18 |
| P-value | 0.36 | 0.52 | 0.68 | 0.24 | 0.76 |
| Global strength difference | 0.24 | 0.55 | 0.24 | 0.15 | 1.20 |
| P-value | 0.80 | 0.38 | 0.72 | 0.80 | 0.12 |
| Note: CM: childhood maltreatment; EA: emotional abuse; PA: physical abuse; SA: sexual abuse; EN: Emotional neglect; PN: physical neglect. | | | | | |

| **Table S4**. Permutation results of network comparison test across the CM only group, the both CM and MDD group, and the non-CM/MDD group. | | | |
| --- | --- | --- | --- |
|  | C1C2 | C1C3 | C2C3 |
| Network invariance | 0.25 | 0.16 | 0.22 |
| P-value | 0.17 | 0.82 | 0.63 |
| Global strength difference | 0.29 | 0.97 | 2.72 |
| P-value | 0.59 | 0.01 | 0.01 |
| Note: CM: childhood maltreatment; C1: individuals with only childhood maltreatment; C2: individuals with both childhood maltreatment and depression; C3: individuals without childhood maltreatment or depression; MDD: major depressive disorder. | | | |

**
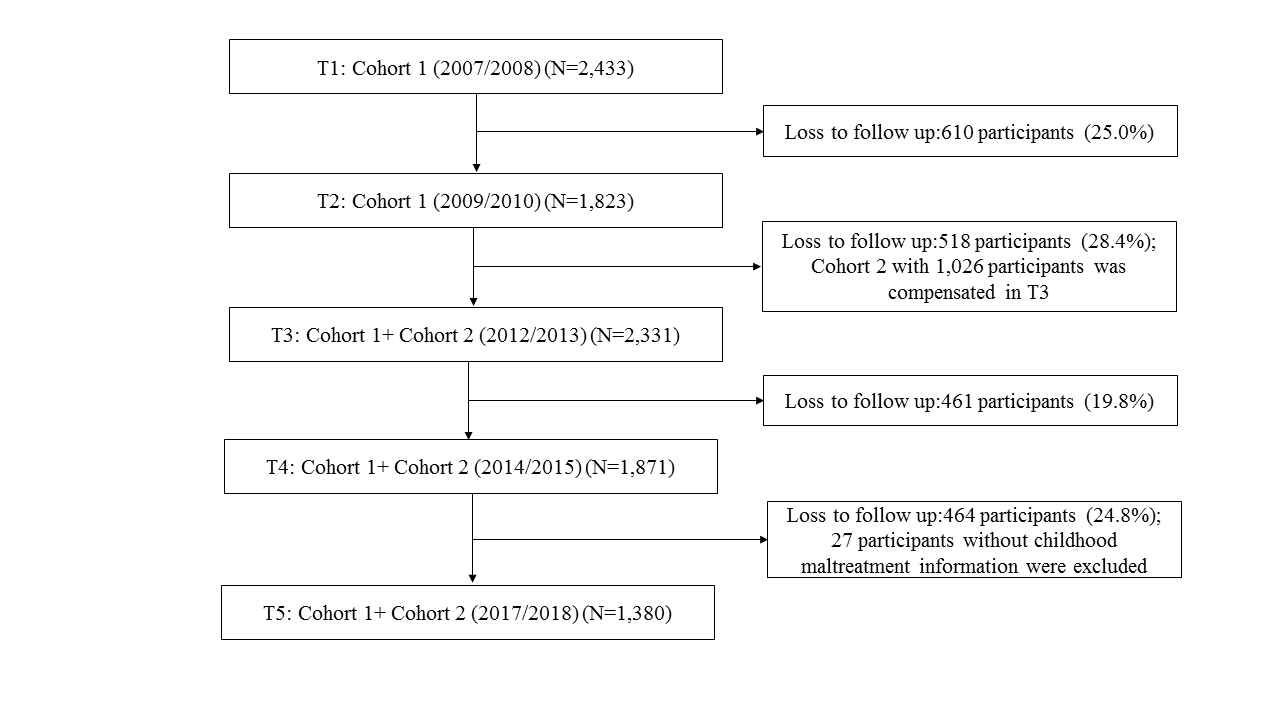
**

**Figure S1**. Flow chart of the study cohort.

**Figure S2**. Centrality indices of study variables within the networks of the maltreatment and the non-maltreatment groups. Note: Centrality indices of node strength are shown as standardized z-scores.


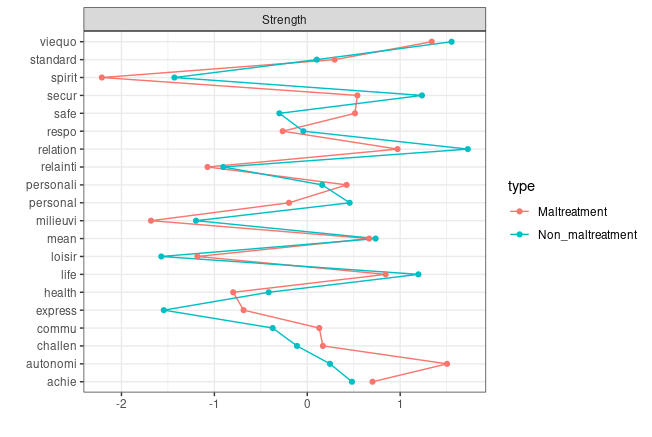


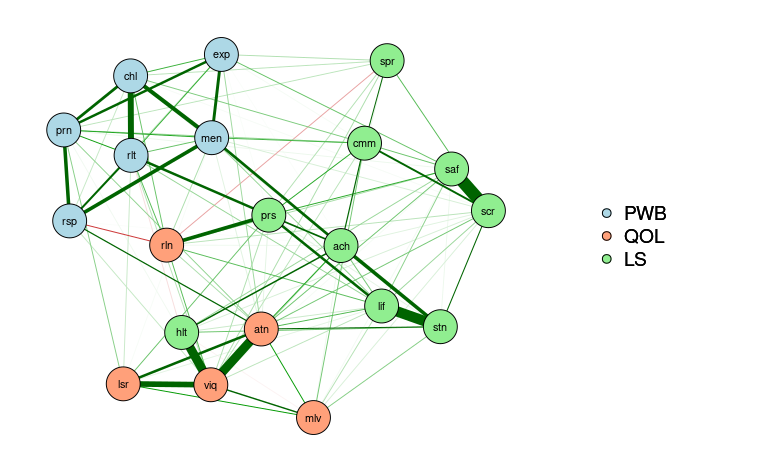

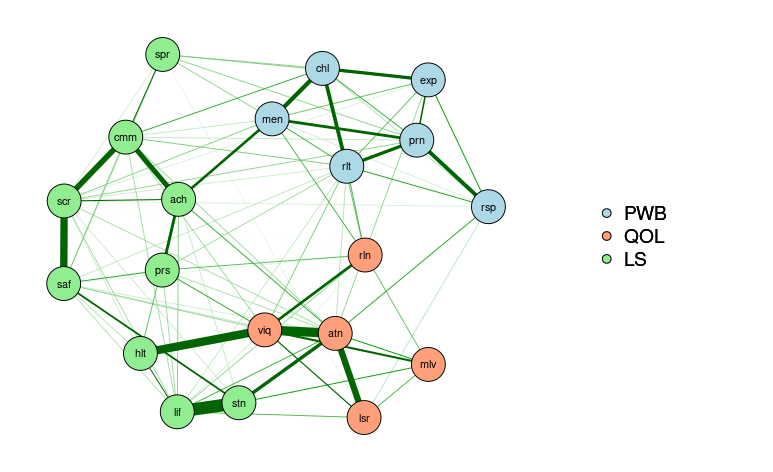
A1) with physical abuse A2) without physical abuse


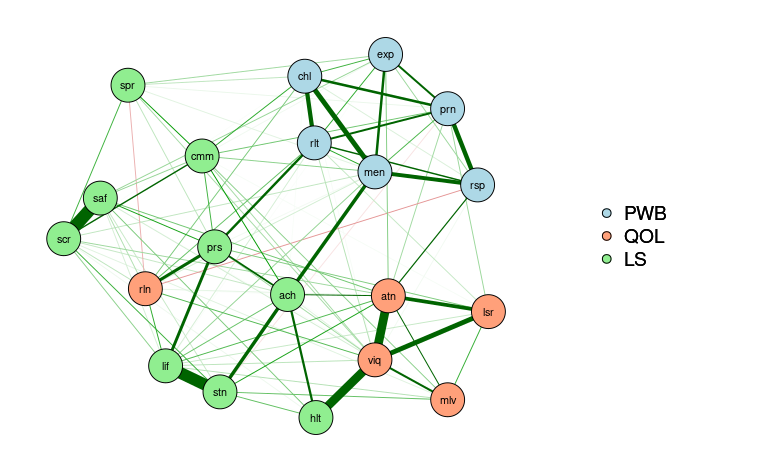

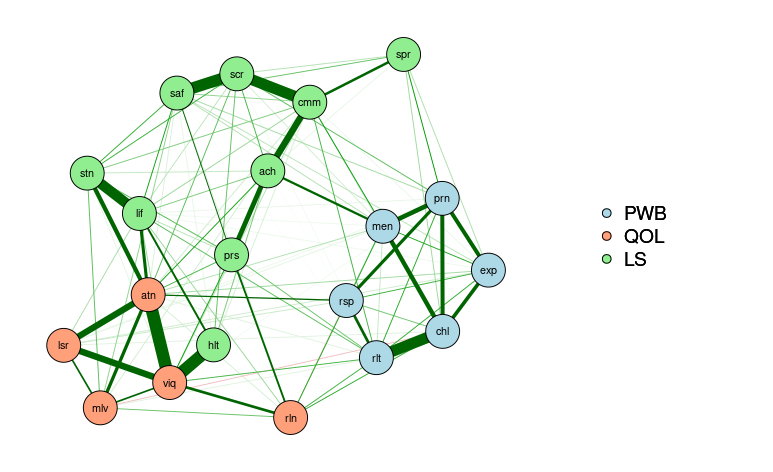
B1) with emotional abuse B2) without emotional abuse


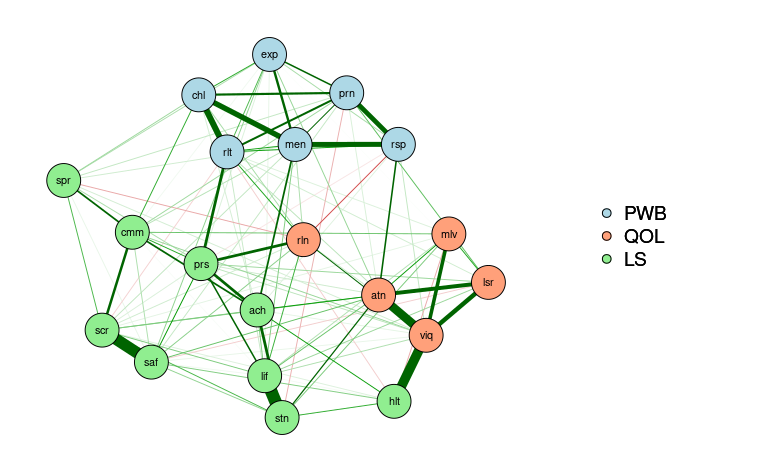

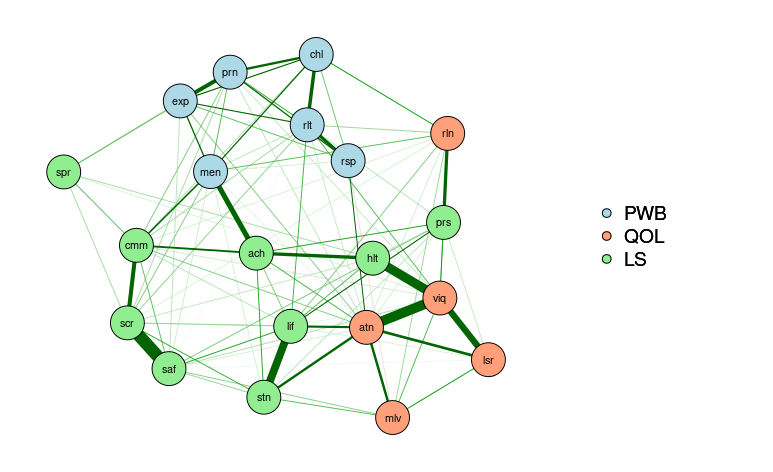
C1) with sexual abuse C2) without sexual abuse


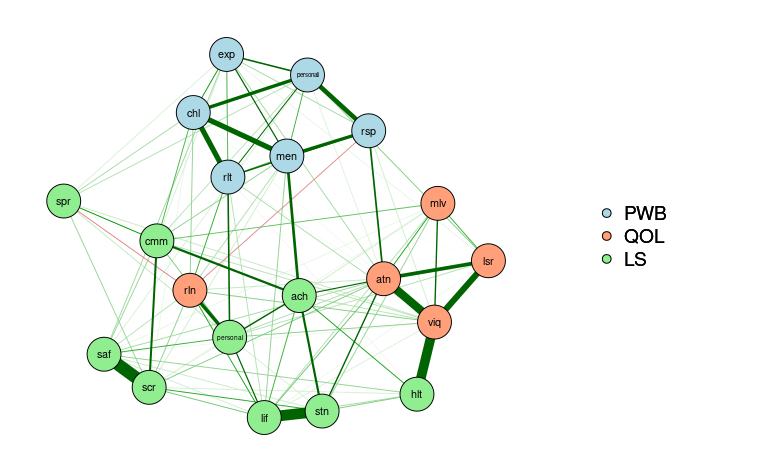

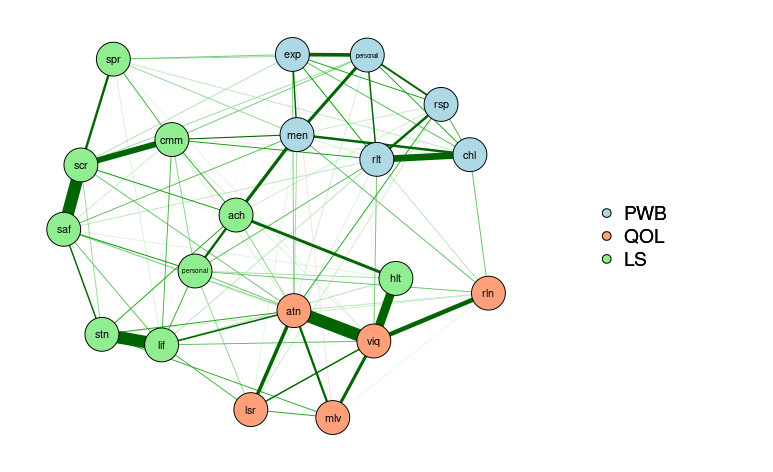
D1) with physical neglect D2) without physical neglect


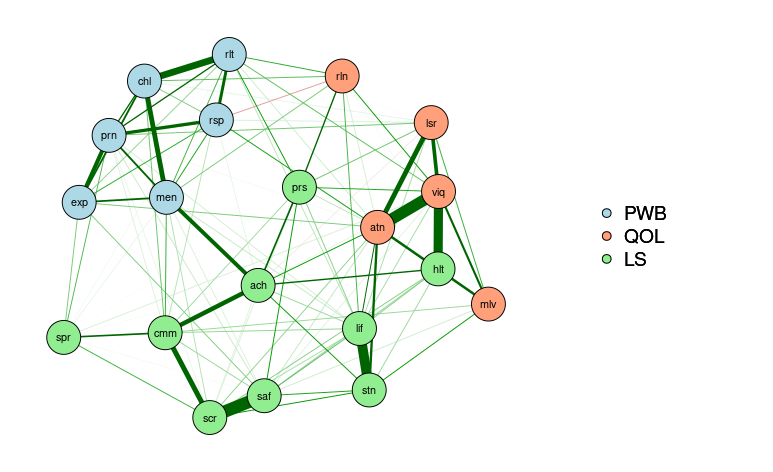

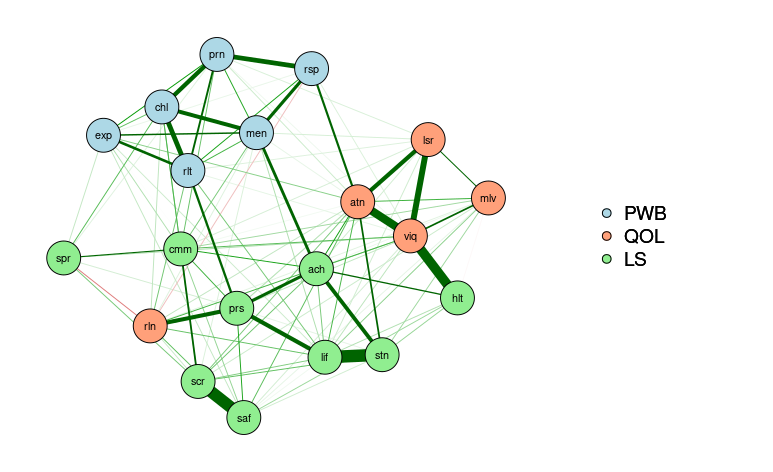
E1) with emotional neglect E2) without emotional neglect

**Figure S3**. The networks of well-being among individuals with and without the exposures to subtypes of childhood maltreatment.

Note: PWB: psychological wellbeing; QOL: quality of life; LS: life satisfaction; prn-liked personality; rsp-responsibilities management; rlt-warm and trusting relationships; chl-challenge for becoming better; exp-express opinions; men-life meaning; mlv-housing-neighbourhood; viq-daily life & social relations; rln-personal relationships; atn- autonomy; lsr-spare time activities; lif-satisfaction with life; stn-satisfaction with living standard; hlt-satisfaction with health; ach-satisfaction with achievement; prs-satisfaction with personal relationship; saf-satisfaction with safety; cmm-satisfaction with community; scr-satisfaction with security; spr-satisfaction with spirituality.

**Figure S4**. Centrality indices of study variables within the networks of the MDD and without MDD groups. Note: Centrality indices of node strength are shown as standardized z-scores; MDD: major depressive disorder.


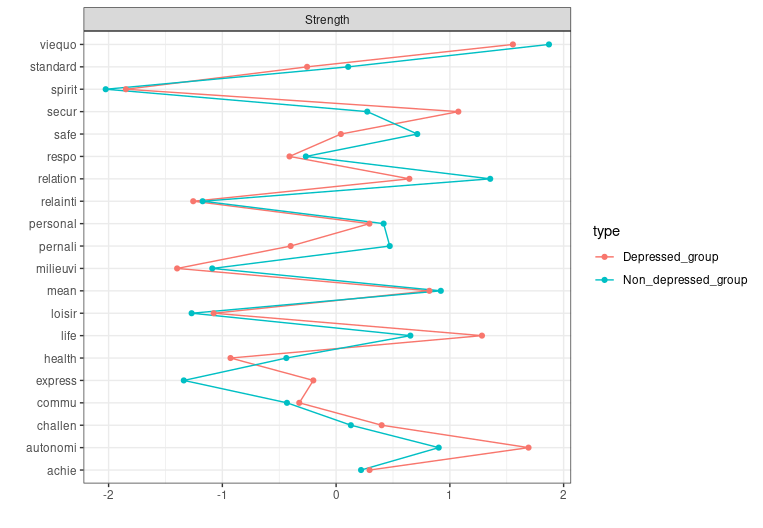


**Figure S5**. Centrality indices of study variables within the networks of the maltreatment only, the both maltreatment and MDD group, and non-maltreatment/MDD groups. Note: Centrality indices of node strength are shown as standardized z-scores. MDD: major depressive disorder.


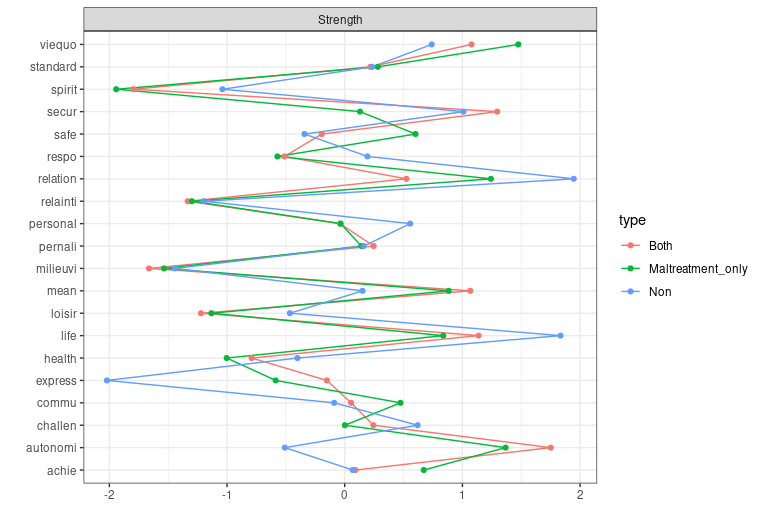

Supplement: Su et al. supplementary material [file S0033291723000673sup001.docx]
